# Muscle mineral profile of water buffaloes (*Bubalus bubalis*) reared in different production systems of the Brazilian Eastern Amazon

**DOI:** 10.3389/fvets.2023.1057658

**Published:** 2023-03-24

**Authors:** Laurena Silva Rodrigues, Jamile Andréa Rodrigues da Silva, José de Brito Lourenço-Júnior, André Guimarães Maciel e Silva, André Martinho de Almeida, Miguel Pedro Mourato, Vinícius Costa Gomes de Castro, Andréia Santana Bezerra, Welligton Conceição da Silva, José António Mestre Prates

**Affiliations:** ^1^Postgraduate Program in Animal Science (PPGCAN), Institute of Veterinary Medicine, Federal University of Para (UFPA), Federal Rural University of the Amazônia (UFRA), Brazilian Agricultural Research Corporation (EMBRAPA), Castanhal, Brazil; ^2^Institute of Animal Health and Production, Federal Rural University of the Amazônia (UFRA), Belem, Brazil; ^3^Linking Landscape, Environment, Agriculture and Food (LEAF), Institute of Agronomy (ISA), University of Lisbon, Lisboa, Portugal; ^4^Postgraduate Program in Animal Health and Production in the Amazon (PPGSPAA), Federal Rural University of the Amazon (UFRA), Belem, Brazil; ^5^Instituto Federal do Pará (IFPA), Institute of Medicine Veterinary, Óbidos, PA, Brazil; ^6^Center for Interdisciplinary Research in Animal Health (CIISA), Faculty of Veterinary Medicine, University of Lisbon, Lisboa, Portugal; ^7^Associate Laboratory for Animal and Veterinary Science (AL4AnimalS), Lisboa, Portugal

**Keywords:** ruminants, ribeye muscle, macrominerals, microminerals, Amazon

## Abstract

Healthy food must have an adequate balance of macroelements, such as calcium or phosphorus and, microelements, such as iron, copper. This study aimed to evaluate the influence of three extensive systems, during the dry and rainy seasons, and an intensive (feedlot) system in the Eastern Amazon, on the muscle mineral profile of water buffaloes. In total, 12 male buffaloes, aged between 24 and 36 months, slaughtered in commercial slaughterhouses, were used in each of the systems considered: Marajó island, Santarém, Nova Timboteua, and a feedlot. Approximately 5 g of muscle was collected, stored, and frozen, until freeze-dried. The samples were analyzed for the mineral profile using inductively coupled plasma-optical emission spectrometry (ICP-OES). There were significant differences (*P* < 0.05) for concentrations of sodium (Na), magnesium (Mg), phosphorus (P), sulfur (S), copper (Cu), zinc (Zn), and iron (Fe). Extensive and intensive systems showed significant differences (*P* < 0.05) for Na, Ca, S, Cu, and Fe concentrations. The season also influenced (*P* < 0.05) K, Ca, P, S, Zn, and Fe concentrations. The location and season of the year had a significant interaction (*P* < 0.05) for K, Mg, P, Zn, and Fe concentrations. The study showed that the different Amazonian production systems and the year season influenced the levels of minerals present in buffalo muscle. The values obtained were, in general, higher in extensive production systems, and Marajó Island stood out with higher mineral values in the dry season. Therefore, the meat from animals reared in these systems is a good mineral source for daily human needs.

## 1. Introduction

Healthy diets must include foods that provide all necessary nutrients in adequate amounts. It must contain carbohydrates, proteins, lipids, and vitamins. In addition, minerals must be present in the food, although in smaller quantities (1, 2). Healthy food must have an adequate balance of macroelements, such as calcium or phosphorus and, microelements, such as iron, copper or, zinc. Some have structural roles (such as calcium and phosphorus), whereas others (such as iron or zinc) are a cornerstone for biological processes namely, enzymatic reactions, cellular osmotic balance, nerve impulse and, muscle contraction (3, 4).

In the Eastern Amazon, water buffaloes are raised in different native pasture-based production systems. These include those of the Marajó island, the Lower and Middle Amazon regions, the drylands (locally known as *terra-firme*), and drylands with agricultural use. The latter type is more productive and has higher nutritional value fodders and thus can be used to feed herds with better genetic and production standards (5).

Animal feed, including pastures, is the primary source of minerals, and their bioavailability will influence the concentration of macro and microminerals in animal tissues (6). Although energy and protein are essential for animal production, it should be stressed that macro and micronutrient deficits over a long season can have drastic effects on animal production and productivity (7).

Numerous factors can lead to changes in mineral concentrations in animal tissues. These include species, gender, genotype, production stage (e.g., lactation cycle), region, climate, tissue characteristics, and animal management and nutrition practices (8–13).

In such context, this study analyses factors that influence the muscle mineral content of buffaloes in the Brazilian Eastern Amazon, namely the production system and the year season. Given the limited number of studies available on water buffalo meat characteristics in this vast region of South America, this study is relevant.

## 2. Materials and methods

### 2.1. Experimental animals

The study was thoroughly described in a companion paper (14). Briefly, we used muscle samples (*longissimus lumborum*) of Murrah × Mediterranean crossbred buffaloes from three different production systems in the Eastern Amazon (under extensive production systems) in two times of the year (dry and rainy seasons) as well as from intensive (feedlot) production system. Per extensive system and season, 12 male buffaloes were used, aged between 24 and 36 months, with an average weight of 432 kg at the end of the rainy season and 409 kg at the end of the dry season. From the feedlot system, only 12 samples were collected, irrespective of the year season. Muscle samples were collected in licensed commercial abattoirs, where the animals were slaughtered, totalling 84 animals.

### 2.2. Production systems

The production systems from which the samples were collected, described in detail by Silva et al. (14), are as follows: System 1—Marajó Island—located in the region of Soure, Pará, Brazil on Marajó Island (latitude 0°39′27.89″ S, longitude 48°42′35.01″O, altitude 7 m). Two distinct seasonal periods were considered: rainy, from January to June (average temperature—AT of 27.7°C, maximum temperature—MaxT of 31.7°C, minimum temperature—MinT of 22.7°C, RH of 87.2% and average rainfall—AR of 316.4 mm), and dry, from September to November (AT of 28.8°C, MaxT of 32.9°C, MinT of 23.3°C, RU of 83.0% and AR of 122.2 mm) (15).

System 2—Santarém—located in the Mesoregion of the Lower Amazon (Baixo Amazonas), Santarém, Pará, Brazil (latitude 02°41′48.83″ S, longitude 54°38′35.43″ W, altitude 108 m). The rainy season occurs between January and May (AT of 26.1°C, MaxT of 29.8°C, MinT of 23.8°C, RH of 87.6% and AR of 296 mm), and the dry season takes place between July, and November (AT of 27.2°C, MaxT of 31.4°C, MinT of 24.2°C, RH of 84.4% and AR of 20 mm) (15).

System 3—Nova Timboteua—located in the Northeast Paraense Mesoregion, Nova Timboteua, Pará, Brazil (latitude 01°12′52.63″ S, longitude 47°24′30.94″ W, altitude 53 m). The pasture area has 825 hectares with 2,000 animals and a stocking rate of 2.42 LU/ha. The dry season occurs from September to November (AT of 29.1°C, MaxT of 33.5°C, MinT of 22.0°C, RU of 75.2%, and AR of 25.1 mm), and a rainy season from December to August (AT of 27.2°C, MaxT of 31.9°C, MinT of 22.5°C, RU of 86.1%, and AR of 259.7 mm) (15).

System 4: Feedlot—rural property located in the Pará Northeast Mesoregion, Tomé-Açu, Pará, Brazil (latitude 02°25′08″ S, longitude 48°09′08″O, altitude 45 m). In this system, the animals entered the feedlot at 12 months of age and were slaughtered after 192 days of feeding.

### 2.3. Feed sampling and bromatological analysis

Feed sampling and bromatological analysis methodology were described in detail by Silva et al. (14).

### 2.4. Tissue sample collection

Tissue sample collection methodology was reported detail by Silva et al. (14).

### 2.5. Sample preparation and digestion for minerals analysis

Approximately 1 g of muscle (LL muscle) was weighed and dried to constant weight at 65°C. After drying, all samples were ground with a Willey grinder and then weighed into a digestion tube (50 ml) to a weight of ~0.3 g.

Sample dissolution was an adaptation of the method described by Ribeiro et al. (16). Briefly, 3 ml of concentrated nitric acid and 10 ml of acid hydrochloride were added to each digestion tube. The samples were left in the acids for 16 h, and 1 ml of hydrogen peroxide was added immediately before digestion to avoid sample loss due to the reaction between the latter and the acids.

After acid mixture addition, the tubes were randomly distributed on a digestion plate (DigiPREP MS, SCP Science, Baie-D'Urfe, Canada) and heated following the pattern: 1 h to reach 95°C and then 1 h at 95°C. After a total digestion time of 2 h, the samples were allowed to cool in a ventilated chamber.

Once at room temperature, samples were diluted with distilled water in a volumetric flask (25 ml). The diluted samples were filtered in sealed vials using filter papers with a diameter of 90 mm (Filter-Lab ref. 1242, Filtros Anoia, Sant Pere de Riudebitlles, Spain). Once ICP-OES readings could occur, the filtered solution was transferred to the ICP tubes. These tubes were then arranged with their respective blanks on a carrier. Each carrier corresponded to one digestion set.

### 2.6. Inductively coupled plasma-optical emission spectrometry readings

ICP-OES readings were performed on a Thermo Scientific ICP-OES iCAP 7200 duo (Waltham, MA, USA) spectrometer equipped with an autosampler. Several elements standards create the calibration curves needed to quantify the different minerals. The detection and quantification of various elements occurred overnight to detect the following elements: Sn (tin), V (vanadium), Li (lithium), Ba (barium), Se (selenium), As (arsenic), Co (cobalt), Zn (zinc), Fe (iron), Mn (manganese), Cu (copper), Pb (lead), Cd (cadmium), Ni (nickel), Cr (chromium), S (sulfur), P (phosphorus), Mg (magnesium), Ca (calcium), K (potassium), and Na (sodium). No further dilution was required for any element before analysis.

### 2.7. Statistical analysis

The experimental design was a completely randomized 3 × 2 + 1 factorial arrangement (three production systems, two seasonal periods, and an additional treatment corresponding to the confinement in the feedlot). The variables were analyzed in the PROC MIXED of SAS version 9.1 (2014) (17) (SAS Institute Inc., Cary, NC, USA). More detail about statistical model and factors explanations can be found in Silva et al. (14).

## 3. Results

Bromatological and mineral values of the diets fed to crossbred buffaloes raised extensively in three types of production systems during the dry and rainy seasons or intensively in feedlots can be found in Silva et al. (14). Macromineral and micromineral profiles are presented in Table 1.

**Table 1:** Mineral composition (mg/kg) in the ribeye of crossbred buffaloes raised extensively in three types of production systems, during dry (DS) and rainy (RS) seasons, or intensely in feedlot, in the Eastern Amazonia (Brazil).

	**Extensive**						**Intensive**			***P*** **-values**		
	**Santarém** ^1^		**Marajó** ^2^		**Nova Timboteua** ^3^		**Feedlot** ^4^	**SEM**	**Extensive vs. intensive**	**Location (L)** ^*^	**Season (P)** ^**^	**L** *P
	**DS**	**RS**	**DS**	**RS**	**DS**	**RS**						
**Macrominerals (mg/kg/ms)**												
Na	1,868.45^bc^	1,819.35^c^	2,208.74^ab^	1,883.27^bc^	2,015.03^abc^	2,030.71^abc^	2,308.40^a^	79.260	0.0002	0.0359	0.0869	0.1028
K	12,209.0^c^	15,844.0^a^	15,066.0^a^	14,404.0^ab^	13,163.0^bc^	15,708.0^a^	14,606.0^AB^	411.980	0.6437	0.2070	<0.0001	<0.0001
Ca	601.11^a^	458.62^b^	619.21^a^	520.29^ab^	522.29^ab^	511.02^ab^	491.77^B^	24.591	0.0824	0.1267	0.0003	0.0590
Mg	930.34^b^	976.38^b^	1,065.67^a^	1,026.79^a^	931.48^b^	970.18^b^	968.45^B^	11.718	0.2413	<0.0001	0.1278	0.0010
P	7,385.22^c^	8,165.45^ab^	8,372.11^a^	8,290.05^a^	7,504.87^c^	8,061.17^ab^	7,870.94^b^	80.403	0.2940	<0.0001	<0.0001	<0.0001
S	7,366.19^a^	7,249.63^ab^	7,293.22^a^	6,863.78^c^	7,322.16^a^	7,083.84^abc^	7,012.67^bc^	62.047	0.0079	0.0031	<0.0001	0.0575
**Microminerals**												
Cu	4.19^b^	4.27^b^	4.12^b^	4.09^b^	4.78^ab^	5.80^a^	3.78^b^	0.321	0.0312	0.0025	0.2217	0.2729
Zn	130.31^c^	183.75^ab^	176.01^b^	164.08^b^	207.07^a^	211.97^a^	165.29^b^	6.676	0.0649	<0.0001	0.0074	<0.0001
Fe	71.43^c^	94.74^a^	97.93^a^	89.08^ab^	77.93^bc^	98.61^a^	78.20^bc^	3.741	0.0153	0.0162	0.0002	<0.0001
Mn	1.83	1.52	2.07	2.08	1.75	1.84	1.44	0.270	0.1668	0.2679	0.7437	0.7223
**Other**												
Ba	4.91	6.90	5.40	5.18	5.88	6.38	5.66	0.54	0.8366	0.2710	0.0877	0.1091

SEM, standard error of the mean.

* Production systems.

** Season periods. Different letters in subscript indicate significant differences in the same row (*p* < 0.05). Na, sodium; K, potassium; Ca, calcium; Mg, magnesium; P, phosphorus; S, sulfur; Cu, copper; Zn, zinc; Fe, iron; Mn, manganese; Ba, barium. Diets = ^1^System Santarém - In this breeding ecosystem, buffaloes are raised in the traditional way on pasture, in native pastures in areas subject to flooding. During the collection of pastures, there was also an availability of cultivated grasses *Panicum maximum* cv. Mombasa and *Bhachiaria brizantha*. ^2^System Marajó - In this ecosystem, buffaloes are reared in a traditional system, fed exclusively on pasture, with grasses native to areas subject to flooding, such as *Panicum elephantipes, Leersia hexandra and Hymenachne amplexicaulis*. ^3^System Nova Timboteua - In this ecosystem, the animals feed on cultivated pastures (*Panicum maximum* cv. Mombaça and *Bhachiaria humidicola*), in areas originally forested (*the animals received wet brewery waste during the dry period). ^4^System Feedlot - In this system, the animals entered the feedlot at 12 months of age and were slaughtered after 192 days of feeding.

There was an interaction between the production system and periods of the year (*p* < 0.05) for almost all the evaluated minerals. All macrominerals (Na, K, Ca, Mg, P, and S) in buffalo muscle were significantly (*P* < 0.05) influenced by the production system. The highest Na level was detected in the intensive system (230.71 Na mg/kgDM). The K level was significantly higher in the rainy season at Santarém (15,844.0 K mg/kg DM) and Nova Timboteua (15,708.0 K mg/kg) and in the dry season at Marajó Island. Ca showed higher values in the muscle of buffaloes raised in extensive systems in Santarém and Marajó island (*P* < 0.05). The muscle of buffaloes from Marajó Island showed the highest values of Mg, among the studied systems, for the dry season (1,065.67 Mg mg/kg) and the rainy season (1,026.79 Mg mg/kg). Phosphorus showed the same trend, with the highest values on Marajó Island in both seasons. For sulfur, the highest values were only in the muscle of buffaloes raised in the dry season in all extensive systems.

All microminerals (except Mn) were significantly influenced by the different production systems (*P* < 0.05). The highest Cu content was in the muscle of buffaloes raised in the rainy season at Nova Timboteua (5.80 Cu mg/kg/MS). Significantly (*P* < 0.05) higher Zn values were found in the muscle of buffaloes raised in the Nova Timboteua system in both the dry (207.07 Zn mg/kg/MS) and rainy (211.97 Zn mg/kg) seasons, with the lowest value present in Santarém in the dry season (130.31 Zn mg/kg). The highest values of Fe were in the meat of buffaloes raised during the rainy season in Santarém and Nova Timboteua and in the dry season at Marajó island (*P* < 0.05).

## 4. Discussion

In this study, we found that the production systems in the Amazon, whether extensive or intensive, influence the content of macro and microminerals in buffalo meat. However, in general, the highest values of these minerals were observed in the extensive systems.

One of the factors that may have contributed to such differences was the different types of feed available to these animals in the various production systems, with diets that consisted predominantly of fodder plants for extensive systems and concentrate for feedlot production systems (14). A similar nutrition effect is also found for the concentrations of macro and microminerals in the edible tissues of different domestic and game animals (6).

The extensive *vs*. intensive production system relationship was evident in the higher Na content (2,308.40 Na mg/kg) in the muscle of buffaloes raised in the feedlot production system. It was most likely due to the diet containing a high-performance premix and other components.

The most abundant mineral in meat was K, for all systems, except in the feedlot. However, the muscle of buffaloes raised in pasture production systems in Santarém in the rainy season (15,844.0 K mg/kg), Marajó island in the dry season (15,066.0 K mg/kg), and Nova Timboteua in the rainy season (15,708.0 K mg/kg), showed the highest values of K (Table 1) that may have been influenced by the season. The abundant and constant levels of this element could be explained by the fact that it is a primordial intracellular ion to provide muscle stimuli (18). Potassium was also the most abundant mineral in ovine muscle, with concentrations exceeding 10,000.0 mg/kg DM, regardless of breed or nutritional level (16).

An adult human requires ~1,200 mg/day of Ca meet their body requirements (19). In this study, it was found higher values in Santarém (601.11 Ca mg/kg) and Marajó island (619.21 Ca mg/kg) production systems in the dry season. Therefore, human need is almost fulfilled when they eat meat from buffaloes raised in these production systems. On the other hand, lower Ca contents (28.3 mg/kg) were detected in beef *longissimus lumborum* muscle (20). In addition, 26.20 mg/kg of was also detected in the muscle of buffaloes from Iran (21). It is also important to note that the year season influenced the different Ca contents in buffalo meat.

The highest levels of Mg were in the muscle of pasture-raised buffaloes on Marajó Island. These values were not influenced by season (dry season 1,065.67 Mg mg/kg, and rainy season 1,026.79 Mg mg/kg muscle) but were by the study area (*P* < 0.05) (Table 1). Then, these meat contents could provide adequate amounts of Mg to adult daily requirements (420 mg) (19). In a survey of the meat nutritional composition from Hereford and Braford cattle finished on pasture or in a feedlot in southern Brazil, it was observed that pasture-finished steers had higher Mg (*P* < 0.001) and lower K (*P* = 0.001) compared to feedlot-finished steers (22).

The P levels were influenced by the season and location, except in Marajó Island, which showed higher *P*-values when compared to other studied production systems for dry (8,372.11 P mg/kg) and rainy seasons (8,290.05 P mg/kg). Muscles from domestic male cattle produced under tropical conditions showed lower P contents of 2,100.5 mg/kg (20) when compared to this study. For avoiding problems with phosphorus deficiency, an adult requires around 700 mg/day of this element (19), which can be supplied by meat buffalos from production systems in Amazon.

The year season influenced the levels of S observed in the muscles of buffaloes raised in the different production systems studied. All the systems showed similar and higher values than those obtained in the rainy season or feedlot (Table 1).

Ruminant meat is a substantial source of protein, Fe, Zn, and Se (selenium) (23). The microminerals (Cu, Zn, and Fe) obtained in the muscle of buffaloes raised in different Amazon production systems are essential for life (9). The season of the year did not influence the results obtained in the muscle of buffaloes reared in these production systems for Cu. However, the study location did. The highest value was in animals from Nova Timboteua production system during the rainy season (5.80 Cu mg/kg) (Table 1).

The muscle of animals from the Nova Timboteua production system, in the dry and rainy seasons, showed the highest levels of Zn and were significantly (*P* < 0.05) different from the other systems studied, which showed the lowest levels of this mineral. These results were influenced by the study place (Table 1). In Brazil, an analysis of *longissimus dorsi* mineral content of cattle finished on pasture or feedlot noticed the increased concentration of Zn in pasture systems in Hereford (37.80 mg/kg) and 1/4 Braford steers (39.50 mg/kg) when compared to 3/8 Braford steers (30.20 mg/kg) (22). In another research work, the mineral composition of beef lamb muscle finished on pasture in New Zealand and a feedlot in the United States, Zn was more representative in the foreleg of New Zealand lambs with a concentration of 62.40 Zn mg/kg (8). In the present work, higher values were found in all studied systems (130.31–211.97 mg/kg). Zinc is an essential element in a series of biological processes, primarily for the correct performance of the immune system since there is a direct interaction with the cells of the immune system (4).

Regarding the values of Fe present in the muscle of buffaloes, the relation between extensive and intensive production systems, the location, the year season, and the location and year season interaction influenced (*P* < 0.05) the results obtained. The highest Fe contents were detected in the muscle of pasture-raised animals in the systems of Santarém in the rainy season (94.74 Fe mg/kg) in the rainy season, Marajó Island in the dry season (97.93 Fe mg/kg) and Nova Timboteua in the rainy season (98.61 Fe mg/kg) of the year (Table 1). Iron content was higher for 1/4 Brafords than purebred Herefords and 3/8 Brafords (*P* = 0.007) in an experiment on pasture-raised and confined animals (22). Buffalo muscle can supply the adult daily need of Fe (18 mg/day) (19) since it has higher values than these reference values.

Chromium (Cr) and nickel (Ni) showed values lower than 2.0 mg/kg in dry matter (DM), whereas arsenic (Ar) and lithium (Li) showed values lower than 1.5 mg/kg. Lead (Pb), cobalt (Co), tin (Sn), and vanadium (V) were < 1.0 mg/kg. Finally, cadmium (Cd) concentrations were below 0.05 mg/kg DM in the muscle. In tropical countries like Brazil, there is a strong pasture seasonality. Therefore, during the dry season, in most cases, there is low availability of plant biomass, in addition to a deficit of nutrients, such as low levels of protein, soluble carbohydrates, and plant mass lignification. Such seasonality requires the producer to pay more attention to the nutrition of animals raised exclusively on pasture, as forage quantity and quality oscillations can cause mineral imbalances throughout the year. However, the abundance of water, as often happens in the rainy season of the year in some regions of Brazil, can penalize the mineral content of the soil. In sandy soils, for example, nutrient leaching may occur as a result of heavy rainfall, which will consequently cause forage in pastures to have a low content of minerals available to incorporate, which will affect the entire food chain (24, 25).

Animal production systems can influence reproductive and productive aspects due to animal welfare índices (26–28), in addition to these factors, the mineral profiles of animal tissues can influence the production capacity due to the differences in nutritional management in an extensive and intensive regime systems. Another factor that should be highlighted is the physical aspect of the activity, the soil where the animals graze, the species of pastures and activities performed by grazing animals. The latter deserves mention, because the more intense the activity developed by the animal, the greater the need for proteins present into Fe, such as myoglobin (29–33).

Although in the Pará state, the dry and rainy seasons of the year are very marked, we did not notice any deficit in the mineral content of the muscles of buffaloes in any of the production systems studied. However, the mineral content of animals raised on the island of Marajó in the dry season was higher. It may occur because the animals in this system remained close to the flooded land, which may have favored the result of the minerals in their muscles. The local characteristics can also justify the higher mineral content. Indeed, during the dry season, when the waters recede and the rivers dry up, the flooded lands (*várzeas*) emerge. Under such conditions, river sediments, rich in nutrients and, therefore, more fertile, are deposited, thus providing more nutritious grass shoots, making this system a better-quality pasture in the dry season.

## 5. Conclusion

The different Amazonian production systems influenced the values of sodium (Na), magnesium (Mg), phosphorus (P), sulfur (S), copper (Cu), zinc (Zn), and iron (Fe), contained in the muscle of buffaloes in the present study. The relation between extensive and intensive production systems showed differences for Na, Ca, S, Cu, and Fe levels. The season of the year influenced the contents of potassium (K), Ca, P, S, Zn, and Fe. The experimental place and the season of the year interaction showed differences for the values of K, Mg, P, Zn, and Fe. The study showed that the different Amazonian production systems and the year season influenced the levels of minerals present in buffalo muscle. The values obtained were, in general, higher in extensive production systems, and Marajó Island stood out with higher mineral values in the dry season. Therefore, the meat from animals reared in these systems is a good mineral source for daily human needs.

## Data availability statement

The raw data supporting the conclusions of this article will be made available by the authors, without undue reservation.

## Ethics statement

The animal study was reviewed and approved by Ethics Committee on the use of animals (CEUA—Comissão de Ética no Uso de Animais) of the Universidade Federal Rural da Amazônia (protocol number: 4542190820). Written informed consent was obtained from the owners for the participation of their animals in this study.

## Author contributions

Conceptualization, data curation, and writing-original draft preparation: JS and LR. Methodology: JS, LR, VC, WCS, and AGMS. Software: LR and JP. Validation: JS, LR, JP, AGMS, and AA. Formal analysis: JS, LR, and JP. Investigation: JS, LR, VC, WCS, AGMS, and AB. Resources, supervision, project administration, and funding acquisition: JP, AA, and JL-J. Visualization: AB. Writing-review and editing: All authors. All authors have read and agreed to the published version of the manuscript.
